# Highly Specific Blood-Brain Barrier Transmigrating Single-Domain Antibodies Selected by an In Vivo Phage Display Screening

**DOI:** 10.3390/pharmaceutics13101598

**Published:** 2021-10-02

**Authors:** Sandra Isabel Aguiar, Joana N. R. Dias, Ana Santos André, Marta Lisete Silva, Diana Martins, Belmira Carrapiço, Miguel Castanho, João Carriço, Marco Cavaco, Maria Manuela Gaspar, Rui Jorge Nobre, Luís Pereira de Almeida, Soraia Oliveira, Lurdes Gano, João D. G. Correia, Carlos Barbas, João Gonçalves, Vera Neves, Frederico Aires-da-Silva

**Affiliations:** 1 Centro de Investigação Interdisciplinar em Sanidade Animal (CIISA), Faculdade de Medicina Veterinária, Universidade de Lisboa, Avenida da Universidade Técnica, 1300-477 Lisboa, Portugal; saguiar@fmv.ulisboa.pt (S.I.A.); joananrdias@fmv.ulisboa.pt (J.N.R.D.); ana.andre@fmv.ulisboa.pt (A.S.A.); marta.lisete.silva@gmail.com (M.L.S.); dlfm94@gmail.com (D.M.); belmira@fmv.ulisboa.pt (B.C.); 2 Instituto de Medicina Molecular-João Lobo Antunes, Faculdade de Medicina, Universidade de Lisboa, Avenida Professor Egas Moniz, 1649-028 Lisboa, Portugal; macastanho@medicina.ulisboa.pt (M.C.); jcarrico@medicina.ulisboa.pt (J.C.); mcavaco@medicina.ulisboa.pt (M.C.); veraneves@medicina.ulisboa.pt (V.N.); 3 Research Institute for Medicines (iMed.ULisboa), Faculdade de Farmácia, Universidade de Lisboa, Avenida Professor Gama Pinto, 1649-003 Lisboa, Portugal; mgaspar@ff.ulisboa.pt (M.M.G.); jgoncalv@ff.ulisboa.pt (J.G.); 4 ViraVector, Institute for Interdisciplinary Research (III), Center for Neuroscience and Cell Biology (CNC), University of Coimbra, Rua Larga, 3004-504 Coimbra, Portugal; rui.jorge.nobre@gmail.com (R.J.N.); luispa@ff.uc.pt (L.P.d.A.); 5 Center for Neuroscience and Cell Biology (CNC), Faculty of Pharmacy, University of Coimbra, Polo das Ciências da Saúde, 3000-548 Coimbra, Portugal; 6 Technophage SA, Avenida Professor Egas Moniz, 1649-028 Lisboa, Portugal; soraia.3oliveira@gmail.com; 7 Centro de Ciências e Tecnologias Nucleares, Departamento de Engenharia e Ciências Nucleares, Instituto Superior Técnico, Universidade de Lisboa, Estrada Nacional 10 (Km 139.7), Bobadela, 2695-066 Boticas, Portugal; lgano@ctn.tecnico.ulisboa.pt (L.G.); jgalamba@ctn.tecnico.ulisboa.pt (J.D.G.C.); 8 Skaggs Institute for Chemical Biology, Department of Molecular Biology, The Scripps Research Institute, 10550 North Torrey Pines Road, La Jolla, San Diego, CA 92037, USA; carlos@scripps.edu

**Keywords:** brain targeting antibodies, blood-brain barrier, in vivo phage display, single-domain antibodies, drug delivery

## Abstract

A major bottleneck in the successful development of central nervous system (CNS) drugs is the discovery and design of molecules that can cross the blood-brain barrier (BBB). Nano-delivery strategies are a promising approach that take advantage of natural portals of entry into the brain such as monoclonal antibodies (mAbs) targeting endogenous BBB receptors. However, the main selected mAbs rely on targeting broadly expressed receptors, such as the transferrin and insulin receptors, and in selection processes that do not fully mimic the native receptor conformation, leading to mistargeting and a low fraction of the administered dose effectively reaching the brain. Thus, there is an urgent need to identify new BBB receptors and explore novel antibody selection approaches that can allow a more selective delivery into the brain. Considering that in vitro models fail to completely mimic brain structure complexity, we explored an in vivo cell immunization approach to construct a rabbit derived single-domain antibody (sdAb) library towards BBB endothelial cell receptors. The sdAb antibody library was used in an in vivo phage display screening as a functional selection of novel BBB targeting antibodies. Following three rounds of selections, next generation sequencing analysis, in vitro brain endothelial barrier (BEB) model screenings and in vivo biodistribution studies, five potential sdAbs were identified, three of which reaching >0.6% ID/g in the brain. To validate the brain drug delivery proof-of-concept, the most promising sdAb, namely RG3, was conjugated at the surface of liposomes encapsulated with a model drug, the pan-histone deacetylase inhibitor panobinostat (PAN). The translocation efficiency and activity of the conjugate liposome was determined in a dual functional in vitro BEB-glioblastoma model. The RG3 conjugated PAN liposomes enabled an efficient BEB translocation and presented a potent antitumoral activity against LN229 glioblastoma cells without influencing BEB integrity. In conclusion, our in vivo screening approach allowed the selection of highly specific nano-antibody scaffolds with promising properties for brain targeting and drug delivery.

## 1. Introduction

In spite of the major advances in the fields of neuroscience and drug development, the efficacy of many potential therapeutics for treatment of central nervous system (CNS) diseases has been systematically challenged by the low permeability of the blood-brain barrier (BBB) [1,2]. In fact, this physical and metabolic selective barrier between the brain and the systemic circulation, is the main bottleneck in brain-targeted drug development and the most important factor limiting the treatment of major unmet neurodegenerative disorders such as Alzheimer’s, Parkinson’s disease and brain tumors [3,4,5,6,7,8,9]. To overcome this barrier, several strategies have been investigated in the past two decades. One of such approaches is the development of specific antibodies that target endogenous BBB transport mechanisms, such as the receptor-mediated transcytosis (RMT) system. By using this native pathway, the antibody specifically binds BBB receptors, translocating into the brain in a controlled and non-damaging manner as a biological “Trojan Horse”, while carrying therapeutic compounds [4,5,6,7,8,9]. The potential of this strategy for CNS drug delivery has already been well-validated for two targets, the insulin receptor (IR) and the transferrin receptor (TfR). Antibodies towards these two receptors have demonstrated the ability to transport therapeutic drugs across the BBB through RMT [10,11,12,13,14,15,16,17,18,19], validating the potential of this pathway for therapy and diagnosis of neurological diseases. Yet, IR and TfR are not brain specific moieties, being highly expressed in other tissues, and implicated in metabolically crucial cellular functions. As such, antibodies towards these receptors could lead to mistargeting of brain drugs to other sites thus resulting in unwanted side effects and creating safety risks [9,20,21,22,23,24,25,26,27,28]. Moreover, the majority of antibodies developed are IgGs, a class of large molecules which limits their brain accessibility and translocation, having systematically failed to attain sufficient concentrations in the brain side and hampering their therapeutic potential [9,29]. In addition, the IgG uptake by widely expressed Fc receptors, along with their prolonged half-lives, further contributes for its non-specific accumulation that may result in systemic secondary effects [30]. Therefore, there is an urgent need for identification of more selective BBB targets or improved antibodies that can enhance the uptake of therapeutic molecules into the brain with minimized non-specific accumulation. For targeting and drug delivery functions, the only components of the IgG molecule that are necessary are the antibody variable binding domains, the VH or VL. Their small size enables to reach epitopes inaccessible to conventional IgGs [30] which, along with the possibility of controlled drug conjugation engineering, paves the way to new drug delivery strategies to successfully transpose the BBB [9,31]. Moreover, single-domain antibodies (sdAbs) are highly stable moieties, present low immunogenicity, decreased manufacturing cost and their structure allows flexible attachment to neuropharmaceuticals or nanoparticles containing biologically active compounds, making them quite attractive drug delivery vectors [32].

**Figure 1:**
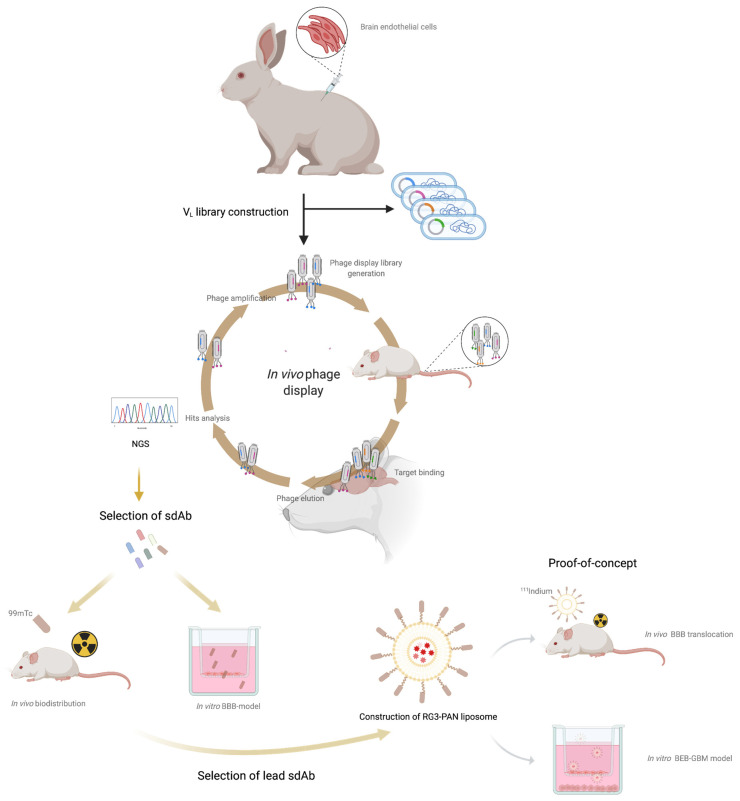
Schematic representation of the in vivo screening process.

In the quest to develop BBB specific antibodies, Muruganandam and collaborators [32] have proposed two promising brain-targeting sdAbs, FC5 and FC44, that were selected by phage display from a camelid VHH library. These two VHHs displayed significant brain accumulation in mice and rapid elimination through kidneys and liver [32]. FC5 was further demonstrated to engage active RMT through the luminal alpha(2,3)-sialoglycoprotein receptor (TMEM30A) [33] and engineered as a FC5-Fc-neuropeptide to validate CNS delivery properties [24,34]. More recently, in a very elegant study, Zuchero et al. performed a detailed transcriptomic and proteomic analysis of mouse brain endothelial cells that allowed the identification of three robust BBB receptors for drug delivery, with translation into the human setting, namely, basigin, glutamate receptor 1 and CD98 heavy chain (CD98hc) [35]. Antibodies developed towards CD98hc presented improved brain targeting and drug delivery properties when compared with the IR and TfR [35]. These studies paved the way to the identification of novel and more promising BBB receptors for biotherapeutic drug delivery into the brain. Nevertheless, these BBB-targets were identified by in vitro selection studies that do not fully mimic the in vivo environment of the BBB and possibly compromise the expression levels and conformation of its native receptors. Indeed, if we look into the in vivo BBB characteristics, it is known that BBB endothelial cells get stimulated by their surrounding cells and intraluminal blood flow [36,37]. This regulates the expression of specific receptors at the cell surface in a polarized fashion contributing to the complexity of the BBB. For these reasons, screening for highly selective BBB transmigrating antibodies should preferably be performed in vivo. As such, in the present study we explored the potential of a whole cell in vivo immunization approach in rabbits followed by an in vivo phage display selection in a murine model aiming to develop potent BBB transmigrating nano-antibody scaffolds, as illustrated in Figure 1. To achieve this, a rabbit derived immunized sdAb library towards brain endothelial cells receptors was constructed and brain specific nano-antibodies were recovered in an in vivo phage display assay. With this combinatorial approach we successfully identified a panel of novel BBB crossing sdAbs that can specifically target and reach the brain. In order to evaluate the potential of our selected sdAbs for CNS drug delivery, the most promising lead antibody was engineered at liposomes surface encapsulating a model drug that has been demonstrated not to cross the BBB, the pan-histone deacetylase inhibitor (HDACi) panobinostat (PAN) [38], and its BEB transmigrating properties and anti-tumor activity were evaluated in a dual functional in vitro BEB-glioblastoma model.

## 2. Materials and Methods

### 2.1. Rabbit Immunization

All animal-handling procedures were performed according to EU recommendations for good practices and animal welfare and were approved by the Animal Care and Ethical Committee with reference FMV_CEBEA_BBB_BIO_0508_2014. The animals were housed in a temperature and humidity-controlled room with a 12 h light-12 h dark cycle. Two New Zealand white rabbits (Charles River) were immunized and boosted at days 14, 28, 56 and 70, with mouse brain endothelial cells bEnd.3 cells (ATCC^®^CRL-2299™) to induce a strong and specific immune response against endogenous bEnd.3 receptors. Briefly, bEnd.3 cells were grown until confluency on T175 flasks in Dulbecco’s Modified Eagle Medium (DMEM) media with high glucose and pyruvate (Gibco) supplemented with 10% heat inactivated fetal bovine serum (FBS) (Gibco) in a humidified atmosphere at 37 °C with 5% CO_2_. Before each immunization procedure, blood was collected from the ear vein and 1 × 10^6^ bEnd.3 cells, suspended in 0.5–1 mL of sterile phosphate buffer saline (PBS), and injected subcutaneously in the rabbit. The injections were administered at 2–3-week intervals. Five days after the final boost, the rabbits were sacrificed by cardiac puncture exsanguination, following propofol anesthesia, and spleen and bone marrow were harvested for total RNA isolation and cDNA synthesis.

### 2.2. Characterization of Rabbit Immune Response

The rabbit immune response developed against the bEnd.3 cells was monitored by ELISA sera testing of the bleeds taken before and after each boost injection. Briefly, 2 × 10^4^/well bEnd.3 cells were plated in a 96 well plate and incubated for 24 h at 37 °C in a humidified environment with 5% CO_2_. In the following day, cells were blocked with PBS-BSA 1% (BSA, bovine serum albumin, Merck, Kenilworth, NJ, USA) for 30 min, washed with PBS and incubated with serial dilutions of the rabbit serum (from 1/500 to 1/32,000) for 1 h. Cells were then washed with PBS and secondary antibody goat-α anti-rabbit IgG-Fc specific HRP (Jackson ImmunoResearch, West Grove, PA, USA) at 1:3000 in PBS-BSA 1% was added to each well and incubated for 1 h. Following incubation, ABTS substrate solution (Merck) was added, and optical density (OD) was measured in a microplate reader (Bio-rad, Hercules, CA, USA) at 405 nm. Each serum was also analysed for its binding profile against bEnd.3 proteins extracts by WB. Briefly, bEnd.3 total protein extracts, obtained after RIPA cells lysis buffer (50 mM tris-HCl pH 7.4; 150 mM NaCl; 1% NP-40, 0.25% Na-deoxycholate), were separated by 11% SDS-PAGE and transferred into PVDF membranes as previously described [39]. Then, WB was performed with each serum at 1/500 dilution in PBS-BSA 1% followed by the goat-(alpha)-anti-rabbit IgG-Fc specific HRP. In addition, the BBB transmigration properties of each serum were evaluated in vitro and in vivo. For that, each serum was purified by protein A chromatography as previously described [40] and then its BBB crossing properties were evaluated as described below in the in vitro BEB model and in vivo section.

### 2.3. Construction of Single-Domain Antibody Library 

Total RNA was extracted from spleen and bone marrow of each rabbit using Trizol reagent (Invitrogen, Waltham, MA, USA) according to the manufacturer’s instructions. First-strand cDNA was synthesized using the transcriptor first strand cDNA synthesis kit (Roche, Basel, Switzerland). The first-strand cDNAs from each rabbit were then subjected to separate 30-cycle polymerase chain reactions using Phusion High Fidelity DNA polymerase (Thermo Fisher Scientific, Waltham, MA, USA) and 10 specific oligonucleotide primer combinations for the amplification of rabbit sdAbs in the format of the V_L_ (9 × V_κ_ and 1 × V_λ_) as previously described [41,42]. The PCR products were purified, digested with *Sfi*I restriction enzyme (Roche), and cloned into the appropriately cut phagemid vector pComb3X [42,43]. The recombinant phagemid was introduced into competent *Escherichia coli* ER2738 (Lucigen, Middleton, WI, USA) cells by electroporation and phages displaying the V_L_ sdAb library were produced as previously described [39] and used immediately in the in vivo phage display panning.

### 2.4. In Vivo Phage Display

For the first selection round, three CD1 mice (Charles River, Wilmington, MA, USA) were intravenously injected into the tail vein with 100 µL of phages (~1 × 10^11^ phages/mL) freshly prepared from the immune V_L_ sdAb library. For optimization purposes, phages were allowed to circulate for different time points (2 min, 60 min, 6 h or 24 h) and then the mice were sacrificed, perfused with PBS, and the brain extracted and weighted. Following brain homogenization in 70 µm cell strainers (VWR), cell homogenates were centrifuged at 1500× *g* at 4 °C, 10 min. Then, the supernatant was discarded, the cell pellet resuspended in 2 mL of wash buffer (PBS-0.05% Tween20), mixed with gentle agitation at room temperature for 2 min, and centrifuged at 1500× *g* at 4 °C, 10 min. This wash step was repeated three times for the first selection round and five times for the next rounds. Then the phages were recovered by incubating the homogenized brain cells with 500 µL of freshly prepared trypsin (1 mg/mL) (Gibco, Thermo Fisher Scientific) supplemented with anti-protease (Merck) and DNAse (1 U/µL) (Invitrogen) for 15 min at 37 °C Then, the eluted phages were recovered after a centrifugation at 14,000× *g* for 10 min at 4 °C and normalized to a final volume of 1 mL in PBS. Each output phage obtained was used for phage titration and re-amplification in ER2738 for a new round of in vivo selection. Phages were also titered in blood. Three rounds of in vivo selections were performed for the 2 min and 60 min time points after a pilot study with all the time points described above. An irrelevant naïve rabbit V_L_ sdAb library and the M13 helper phage were also used as controls in the pilot study.

### 2.5. Analysis of In Vivo Phage Display Enrichment and Next Generation Sequencing

To analyse the enrichment and profile obtained after each round of in vivo phage display selection, individual clones from the initial V_L_ sdAb library, second and third selection rounds were randomly chosen and sequenced at Eurofins company. Sequence analysis was performed using the Vector NTI software (Invitrogen) and antibody frameworks, CDRs and the amino acid numbering and sequences alignment were performed as defined by Kabat et al. [44]. Furthermore, to analyse the overall diversity and enrichment obtained, we performed NGS. For that, we used the 250-paired ended module of the MiSeq (Illumina, San Diego, CA, USA) sequencing platform to obtain the whole sequence of the V_L_ sdAb regions selected in the third biopanning for both time points (2 min and 60 min). The Miseq library for DNA sequencing was prepared by amplifying the V_L_ sdAb regions and 2 µg of the purified amplicons were sent to the STABVIDA company for sequencing. The NGS sequence data was analyzed using the Geneious software (Biomatters Ltd., Auckland, New Zealand). Sequence data was processed by merging the paired-ended sequence reads, translating the sequence into protein and discarding all sequences with less than 100 amino acids and no *SfiI* cut site and histidine tail sections. Subsequently, an in-house custom python script was developed to summarize and count the sequence reads. Bar charts representing the pattern of sequence reads were generated.

### 2.6. Expression and Purification of V_L_ sdAbs

To express and purify the selected clones, genes encoding the V_L_ sdAbs were transferred into the pET21a plasmid (Novagen, Birmingham, UK) and transformed into *Escherichia coli* BL21 (DE3) electrocompetent cells (Invitrogen). A fresh colony of each V_L_ sdAb clone was grown overnight at 37 °C in Super Broth (SB) medium containing 100 μg/mL of ampicillin. A 10 mL sample of cells was used to inoculate one liter of SB medium containing 100 μg/mL of ampicillin. Cells were grown at 37 °C until O.D._600nm_ = 0.6, induced with 0.6 mM IPTG and growth was continued for 18 h at 19 °C. After induction, bacteria were harvested by centrifugation (4000× *g*, 4 °C, 15 min) and suspended in 50 mL equilibration buffer (20 mM NaH_2_PO_4_, 500 mM NaCl, 30 mM imidazole, and pH 7.4) supplemented with protease inhibitors (Merck). Cells were lysed by sonication. Centrifugation (14,000× *g*, 4 °C, 30 min) was used to remove cellular debris, and the supernatant was filtered through a 0.2 μm syringe filter. The V_L_ sdAbs were then purified by immobilized metal affinity chromatography (IMAC), using HP Histrap columns and the AKTA Start system (GE Healthcare, Chicago, IL, USA), using the C-terminal His_6_ of pET21a. After a washing step, elution of the V_L_ sdAbs occurred by a linear imidazole gradient from 60 to 300 mM in elution buffer. The eluted fractions were pooled, desalted and concentrated in PBS using 3K Amicon columns (Merck). Then, the V_L_ sdAbs samples were loaded onto a HiPrep 16/60 Sephacryl S-100 HR gel filtration column (GE Healthcare) and pooled fractions were analysed for protein purity by 15% SDS-PAGE followed by Coomassie blue staining and WB with HRP-conjugated anti-His antibody (Roche). The concentration of proteins was determined by measuring the absorbance at 280 nm in the Nanodrop 2000 (Thermo Fisher Scientific). The same procedure was performed to express and purify the control antibody, FC5 VHH.

### 2.7. In Vitro BEB Models

The in vitro BEB models were optimized based on previous studies [45]. Briefly, bEnd.3 cells were cultured in DMEM, supplemented with 10% FBS and 1% penicillin/streptomycin (Lonza, Basel, Switzerland) antibiotic solution. Cells were cultured in a humidified atmosphere of 5% CO_2_ at 37 °C and the medium changed every other day. The cells were adherent in monolayers and when confluent, harvested from cell culture flasks with trypsin EDTA (Gibco) and 4 × 10^3^ cells/well were seeded in 24-well plates tissue culture inserts (Falcon, Atlanta, GA, USA) previously coated with bovine plasma fibronectin (1 mg/mL) (Merck). To allow the formation of tight junction’s cells were incubated for 11–14 days and the media changed every 2 days. To evaluate the integrity of the in vitro BEB model before and during the assay day, a fluorescent probe of fluorescein isothiocyanate-dextran with a MW of 4 kDa (FD4) and 40 kDa (FD40) (Merck) and stock concentration of 25 mg/mL were diluted in transport buffer (TB) (5 mM glucose, 5 mM MgCl_2_, 10 mM HEPES at pH 7.4 and 0.05% BSA) to an O.D._493nm_ of 0.1. Probes were then added to the apical side (apex) of the transwell and incubated for 2 h. Samples were recovered from the apex and base and fluorescence intensity was measured in a microtiter plate reader (BMG Labtech, FLUOstar OPTIMA, Ortenberg, Germany) with an excitation of 485 nm and a maximum emission at 520 nm.

### 2.8. In Vitro BEB Translocation

To determine the *in vitro* BEB translocation efficiency of the selected V_L_ sdAb, 15 μg of each purified antibody was added to the apex of the transwell and incubated for 15 and 90 min. The incubation time and sdAb concentration were selected based on previous optimization assays (data not shown). Following incubation, the cells were washed once with PBS and three times with TB. Evaluation of the translocation efficiency of each V_L_ sdAb was assessed by running 15 μL of the recovered volume from the apex and the base on a 15% SDS-PAGE acrylamide gel followed by WB analysis with HRP-conjugated anti-His antibody at 1:3000 (Roche). As a positive control, 100 ng of each purified V_L_ sdAb was used. The FC5 VHH was used as a positive control. The same procedure was performed to evaluate the BEB crossing properties of each purified rabbit serum, but the incubation was performed for 15 and 60 min and the detection was performed with goat-α anti-rabbit IgG-Fc specific HRP at 1:10,000. Chemiluminescence was detected using the Chemidoc XRS+System (Bio-rad).

### 2.9. Biodistribution Studies

The V_L_ sdAbs selected to proceed to in vivo biodistribution studies were radiolabeled with the radioactive precursor [^99m^Tc(CO)_3_(H_2_O)_3_]^+^, which was prepared by addition of a 0.9% saline solution of Na [^99m^TcO_4_], eluted from a ^99^Mo/^99m^Tc generator, to an IsoLink^®^ kit (Covidien) [31]. The radiochemical purity of the precursor was monitored by reversed-phase high-performance liquid chromatography (RP-HPLC) and instant thin-layer chromatography silica gel (ITLC-SG, Agilent Technologies, Santa Clara, CA, USA). Briefly, a specific volume of the *fac*-[^99m^Tc(CO)_3_(H_2_O)_3_]^+^ solution was added to a nitrogen-purged closed glass vial containing a solution of the His-tag containing sdAb in order to get a final concentration of 1 mg/mL. The mixture reacted for 45–60 min at 37 °C and the radiochemical purity of ^99m^Tc(CO)_3_-sdAb was evaluated by ITLC-SG analysis using a 5% HCl (6 M) solution in MeOH as eluent. The precursors [^99m^Tc(CO)_3_(H_2_O)_3_]^+^ and [TcO_4_]^−^ migrate in the front of the solvent (Rf = 1), whereas the radioactive sdAb ^99m^Tc(CO)_3_-sdAb remains at the origin (Rf = 0). Radioactivity distribution on the ITLC-SG strips was monitored using a miniGita Star scanning device (Raytest, Straubenhardt, DE) coupled with a Gamma BGO-V-Detector (Elysia Raytest, Straubenhardt, Germany). Purification of the ^99m^Tc-labeled sdAb was performed using a 10 K Amicon (Merck Millipore) centrifugal filters for protein purification and concentration as described by the supplier. The filtrate was discarded and the concentrate containing ^99m^Tc(CO)_3_-sdAb was diluted in PBS and used for the biodistribution studies in CD1 mice. The radiochemical purity (>95%) was determined by ITLC-SG. Biodistribution studies of radiolabeled sdAbs were performed as previously described [31,45]. Animals were intravenously injected into tail vein with the corresponding ^99m^Tc(CO)_3_-sdAb (0.2–7.9 MBq) diluted in 100 μL of PBS pH 7.2. Mice were sacrificed by cervical dislocation at 2 and 60 min after injection. The dose administered and the radioactivity in the sacrificed animals was measured using a dose calibrator (Carpintec CRC-15W). The difference between the radioactivity in the injected and the euthanized animals was assumed to be due to excretion. Brain and tissues of interest were dissected, rinsed in PBS to remove excess blood, weighed, and their radioactivity measured using a γ-counter (Berthold, Bad Wildbad, Germany). The uptake was calculated and expressed as a percentage of injected radioactivity dose per gram of organ or tissue (%ID/g).

### 2.10. Measurement of Brain Antibody Concentrations

To validate the in vivo translocation efficiency of each rabbit serum CD1 female mice were injected intravenously in the tail vein with 100 μg of purified antibody. Mice were sacrificed at 2 and 60 min after injection. The incubation time and antibody concentration were selected based on previous optimization assays (data not shown). Following blood recovery, mouse brain, kidney, and liver were isolated and homogenized as described above. The antibodies were recovered from each organ by immunoprecipitation (IP) with Dynabeads protein A pull-down beads (rabbit serum) according to the manufacturer protocol. 15 μL of the IP elution was separated in 15% SDS PAGE gel and WB was performed with 1:3000 diluted conjugated 1:10,000 diluted anti-rabbit-HRP antibody. Chemiluminescence was detected using the Chemidoc XRS+System (Bio-rad).

### 2.11. Development of PAN RG3 and FC5 Functionalized Liposomes

The encapsulation of PAN in liposomes was performed by an active loading method with an ammonium sulphate gradient as previously described [46]. Briefly, the relevant lipids, Dipalmitoyl phosphatidyl choline (DPPC), poly(ethylene glycol) (PEG-2000) covalently linked to distearoyl phosphatidyl ethanolamine (DSPE-PEG), and the functionalized DSPE-PEG phospholipid with biotin (DSPE-PEG-biotin), purchased from Avanti Polar Lipids, at a molar ratio of DPPC: Chol: DSPE-PEG:DSPE-PEG-biotin-1.85:1:0.14:0.01, were dissolved in chloroform and the organic solvent was removed by rotary evaporation. The formed homogeneous lipid film was hydrated with water and the so-formed suspension was frozen (−70 °C) and lyophilized in a freeze-dryer (Edwards, CO, USA) overnight. The rehydration of the lyophilized powder was performed with ammonium sulphate (135 mM, pH 5.4) at 45 °C for 30 min. In order to produce a homogeneous liposomal suspension, unloaded liposomes were filtered under nitrogen pressure (10–500 lb/in^2^), through polycarbonate membranes of proper pore size (at 45 °C), using a Lipex thermo-barrel extruder (Lipex: Biomembranes Inc., Vancouver, BC, Canada) until achieving liposomes with a mean size of around 0.1 µm. An ammonium sulphate gradient was created by replacing the extra liposomal medium with PBS buffer (pH 7.4) using a Econo-pac 10 DG desalting column (Bio-rad). PAN was incubated with unloaded liposomes, at a molar ratio 1:16 µmol of lipid, previously diluted in PBS (from a stock solution of 67 mg/mL) for 60 min at 45 °C. The non-encapsulated PAN was separated by ultracentrifugation at 250,000× *g* for 2 h at 15 °C in a Beckman LM-80 ultracentrifuge (Beckman Instruments, Inc., Fullerton, CA, USA). The pellet was suspended in PBS (pH 7.4). RG3 and FC5 antibodies were biotinylated using the kit EZ-Link™ Sulfo-NHS-LC-Biotinylation Kit” (Thermo Fisher Scientific), with a molar ratio of 1:30 (mole antibody: mole biotin). The biotin-antibody conjugate was mixed with streptavidin at a molar ration of 3:1 (mole antibody/mole streptavidin) at room temperature for 20 min. The mixture was then incubated with the pre-formed biotin-liposomes in a molar ratio of 1:1 (mole biotin in the liposome/mole antibody biotinylated) at room temperature for 2 h and later overnight at 4 °C. Non-attached sdAb was removed by centrifugation using a 100 K Amicon^®^ Ultra-4 membrane filter (Merck). Biodistribution studies of selected RG3-conjugated PAN liposomes were carried out with ^111^In. For that, the chelating agent diethylenetriamine pentaacetic acid (DTPA) at a concentration of 6 µM was encapsulated during liposome preparation after achievement of the lipid film and before lyophilization [47]. RG3 functionalized liposomes co-loaded with DTPA were labelled with ^111^In using the lipophilic complex ^111^In-oxine as precursor. The ^111^In-oxine complex passively crossed the lipid membrane and in the internal aqueous compartment of liposomes transferred the metal ion to DTPA where the hydrophilic complex ^111^In-DTPA remained trapped. Radiolabeling and subsequent biodistribution studies were performed as described above.

### 2.12. Translocation Efficiency Assay of Lip-RG3 and Lip-FC5

Translocation efficiency of each liposome formulation was validated on the in vitro BEB model with bEnd.3 cells as described above. Briefly, empty rhodamine labelled RG3 and FC5 functionalized liposomes were previously diluted in DMEM without phenol red to a final concentration of 1.15 ng/μL (considering the ratio of antibody) and added to the apical side of the in vitro BEB model. The apex volume and the base volume were collected after 90 min, 6 and 24 h, the fluorescence in those samples was measured separately in a microplate reader (Fluostar Optima Bmg Labtech) and the translocation was calculated using the equation: translocation (%) = Fi/Ft × 100, where Fi is the recovered fluorescence intensity at the base and Ft is the fluorescence intensity of the total sdAb added to the apical side of the transwell. To determine the antitumoral effect of Lip-RG3 and Lip-FC5 encapsulated with PAN on LN229 cells, a cell viability assay was performed using the Cell Proliferation Reagent WST-1 (Roche). The cells were seeded at a density of 5 × 10^3^ cells/well in 96-well plates in 200 µL of DMEM culture medium supplemented with 10% FBS and 1% penicillin-streptomycin. The cells were subjected to increasing concentrations of PAN encapsulated liposomes, and the respective controls (free PAN, Lip-PAN, Lip-RG3, Lip-FC5 and empty liposome). After 24 h of treatment, WST-1 reagent was added, following the manufacturer’s instructions, to determinate the cell viability. O.D._450 nm_ was measured in a plate reader following a 24 h incubation with the reagent. Each data point was determined using three replicate wells and two independent experiments. Best-fit IC_50_ values were calculated using GraphPad Prism software (version 9.00; San Diego, CA, USA), using the log (inhibitor) vs. response (variable slope) function.

### 2.13. BEB-Glioblastoma In Vitro Model

To study the BEB translocation efficiency of the Lip-PAN-RG3 and Lip-PAN-FC5 liposomes and subsequent drug delivery and cytotoxic activity of PAN in a glioblastoma cell line, a dual in vitro non-contact co-culture model was established with bEnd.3 and LN229 cells. Briefly, bEnd.3 cells were cultured in tissue culture inserts until confluence as described above. 24 h before the assay day, LN229 cells were cultured in a 24-well plate at a density of 2 × 10^4^ in DMEM medium and the transwell bEnd.3 cells culture were transferred to the 24-well plates. Free and encapsulated PAN loaded RG3 and FC5 functionalized liposomes were added to the apical side of the transwell and incubated for 24 h. Following incubation all volume was recovered from the apex, the transwell was removed and the LN229 cells were incubated further 24 h. Following the 24 h treatment, WST-1 was added to the plate, and O.D._450nm_ was measured following 24 h incubation. Each data point was determined using three replicate wells and two independent experiments were carried out in different days. To confirm that the cytotoxic effects of the BBB targeted PAN liposomes on GBM cell line was related to histone acetylation induction, the protein extract samples were quantified using the Bradford method (Coomassie Plus™ Kit, Thermo Fisher Scientific) according to the manufacturer’s instructions and analysed by WB using anti-acetyl histone H3 (Lys9, Lys14) antibody (polyclonal, rabbit, 1:2500 dilution, Thermo Fisher Scientific), anti-histone H3 (polyclonal, rabbit, 1:1000 dilution, Thermo Fisher Scientific) as primary antibodies and anti-rabbit IgG-Fc specific HRP (polyclonal, goat, 1:10,000 dilution, Jackson ImmunoResearch) as secondary antibody. Protein detection was performed by chemiluminescence using Luminata Forte Western HRP (Merck) and acquired using the ChemiDoc XRS+ imaging system (Bio-Rad). In parallel, the integrity of the BEB model was measured as described previously.

### 2.14. Statistical Analysis

All data was expressed as mean ± standard error of mean (SEM). Analysis was performed using Prism 9 (Graphpad Software). For in vitro assays, statistical significance of results was determined by One-way ANOVA followed by Tukey Multiple Comparison test to compare individual groups. Statistical analysis of the biodistribution data (ANOVA for evaluation of data from labeled sdAbs and *t*-test for data from liposomes) was also done with GraphPad Prism (version 9.00) and the level of significance was set as *p*-value < 0.05.

## 3. Results and Discussion

### 3.1. Construction of the Immunized sdAb Library and Antibody Validation

**Figure 2:**
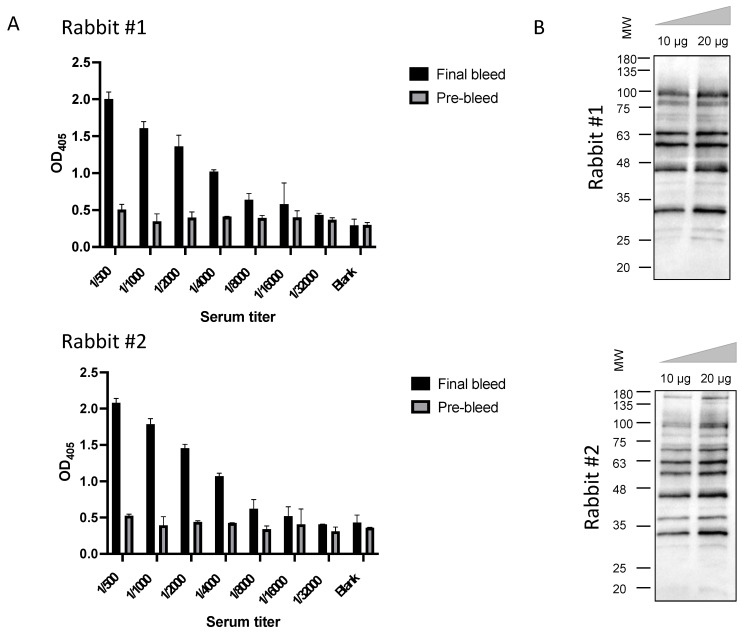
Analysis of rabbit immunological response: (**A**) ELISA serum titration of Rabbit #1 and Rabbit #2 total serum against bEnd.3 endothelial cells. Total rabbit serum was diluted by serial dilution (from 500 to 32,000-fold) and tested in 2 × 10^4^ cells/well. Results showed a selective and specific immune response against bEnd.3 cells. (**B**) Target validation and assessment of potential bEnd.3 receptors recognized by each rabbit serum following immunological boost by WB. Total protein extract was obtained with RIPA buffer from bEnd.3 and 10 μg and 20 μg was loaded in a 11% SDS page acrylamide gel. Following gel transfer and membrane blockage, total rabbit serum 500-fold diluted was added to determine the specificity of the antibodies produced by the rabbit immunization process towards bEnd.3 total protein extract. Results demonstrated that antibodies towards several receptors were developed in both immunized rabbits validating our immunization approach.

To address the complexity of the BBB, a phenotypic antibody discovery approach that considers the native conformation of BBB cell surface receptors was developed, as schematically represented in Figure 1. The nano-antibody sdAb library was constructed by whole-cell immunization of two New Zealand White rabbits with a cell line of mouse brain endothelial cells (bEnd.3). Rabbit antibodies are well known for their ability to produce high affinity and site-specific antibodies, being capable of generating antibodies that recognize similar epitopes from different species [48]. More importantly, contrary to rodents, rabbits develop highly diverse and strong immune responses particularly against low abundant proteins or hidden epitopes, particularly prevalent in the BBB receptome [49,50]. The rational use of the bEnd.3 cell line relates with the subsequent in vivo assays for validation of our immunization strategy. To monitor the rabbit immunological immunization response, bleeds were taken before and after each immunization boosting and evaluated by ELISA (Figure 2A) and Western Blot (WB) (Figure 2B) to confirm the serum specificity and titer against bEnd.3 surface receptors. The results obtained for the sera of the two rabbits showed that the immunization process originated a strong and specific immune response against the BEB cellular model receptors, with no antibody recognition of the bEnd.3 receptors in the pre-immunization serum (data not shown).

**Figure 3:**
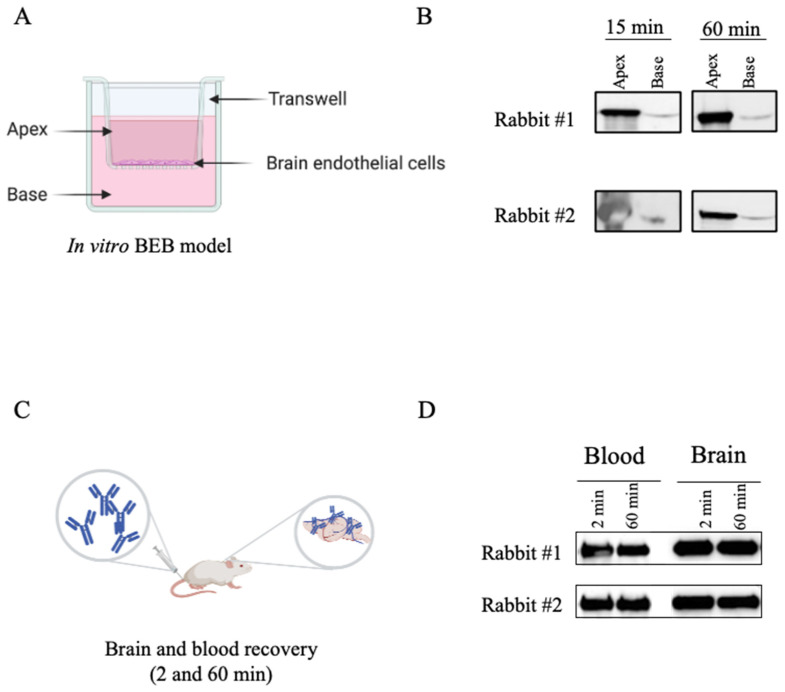
Validation of the rabbit serum BBB crossing features. To confirm that the antibodies obtained from the rabbit immunization process retained the ability to cross the BBB, purified serum from both rabbits were tested in both in vitro and in vivo models. (**A**) For the in vitro BEB model 4 × 10^3^ bEnd.3 cells were seeded in 24-well plates tissue culture inserts and incubated for 11–14 days in a humidified chamber at 37 °C, supplemented with 5% CO_2_. (**B**) To determine the rabbit serum translocation efficiency, 10 µg of purified serum was added to the apex, incubated for 15 and 60 min, recovered from both the apex and the base, and analysed by WB. Rabbit serum was detected at the base of the monocellular model in both time points validating the translocation ability of the rabbit IgGs. (**C**) To confirm the ability to reach the brain in an in vivo model, purified serums (250 µg) were intravenously injected into the tail vein and after 2 and 60 min the mice were sacrificed, and the brain extracted. (**D**) Rabbit immunoglobulins were recovered from the mice blood and homogenized brains by IP with protein A beads and analysed by WB with goat anti-rabbit-HRP IgG at 1:1000. The detection of the rabbit serum in the brain extract validated the ability of rabbit derived antibodies to reach the mice brains and further confirmed the anticipated species cross-reaction.

Considering that our major goal was the identification of antibodies capable of translocating the BBB, we assessed the ability of the final serum from each rabbit to transpose the BBB in a well characterized in vitro BEB model composed of a monolayer of brain endothelial cells (Figure 3A). To determine the translocation properties of the rabbit polyclonal sera, purified final serum was added to the apex and incubated for two time points chosen based on previous optimization assays. As shown in Figure 3B, WB analysis of the rabbit final serum confirmed the presence of antibodies with the ability to translocate into the basal compartment of the monoculture barrier model, with transposition observed at 15 and 60 min. All in vitro BEB model assays were monitored for cellular integrity following antibody incubation, based on the permeability of the barrier to FD40 probe translocation. Paracellular leakage was negligible in all cases (data not shown). Despite its high reproducibility, a major weakness of in vitro BEB models is the decreased complexity in terms of receptor expression when compared to in vivo, which could bias the antibody validation process. As such, to confirm that the rabbit derived antibodies were reaching the brain in a more dynamic model, purified rabbit serum was intravenously injected in CD1 mice (Figure 3C) and, following 2 and 60 min post-injection (p.i.), recovered from the blood and brain by immunoprecipitation with protein A as described in the material and methods section. As demonstrated in Figure 3D, antibodies retrieved from both immunized rabbits were detected in the mice brains, validating the immunization strategy and the presence of antibodies with BBB-crossing features.

### 3.2. In Vivo Phage Display Selection of BBB-Targeting sdAbs

**Figure 4:**
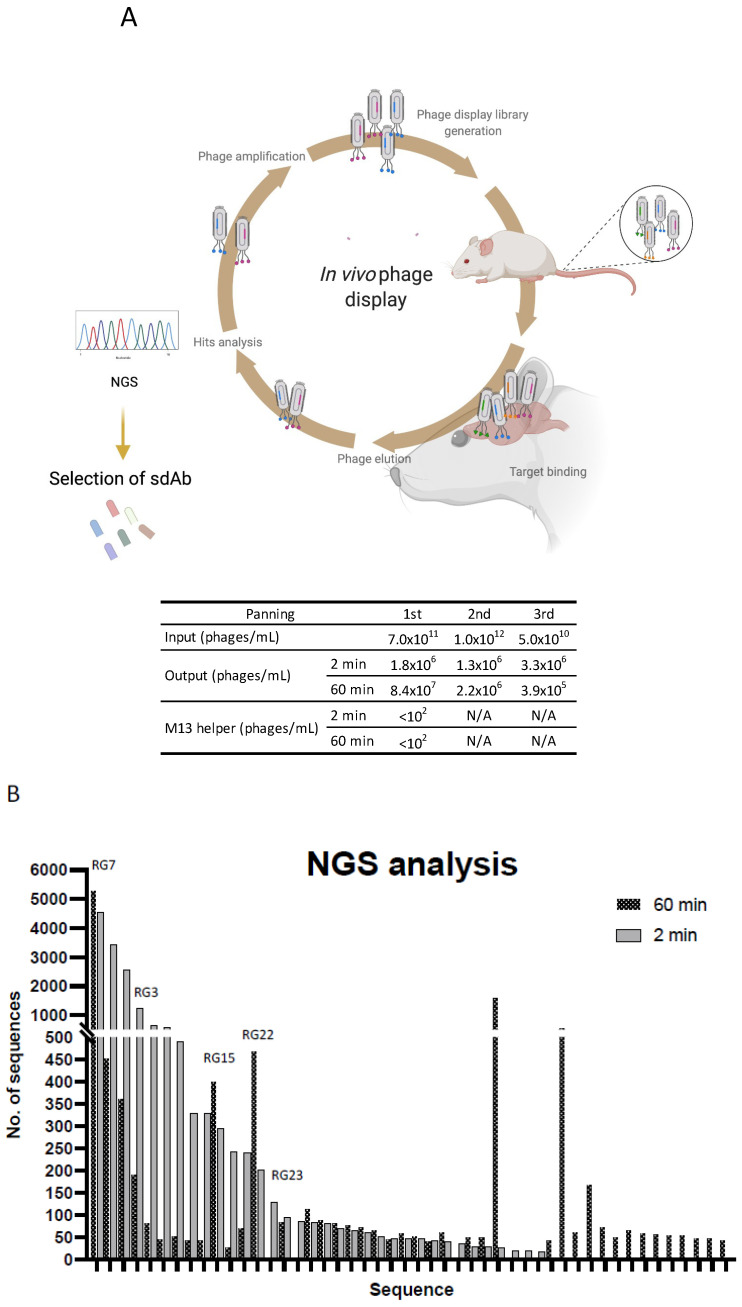
Selection of sdAbs by in vivo phage display and NGS. (**A**) In order to elect the most promising sdAbs for brain targeting and BBB transposition, the rabbit derived immunized phage displayed library was intravenously injected into the tail vein of CD1 mice. Phages were allowed to circulate for 2 and 60 min and the mice were perfused, sacrificed and phages extracted from the brain were re-amplified. Three rounds of in vivo biopanning were performed, in which the phages recovered from the brain were reamplified and re-injected into the mice for a new round of selection. For each selection round, quantification of the injected phages (input) and the phages recovered from the brain (output) was determined. (**B**) Following in vivo selection, the selected phage library was re-amplified and analysed by next-generation sequencing and data analysed by an in-house bioinformatic script. Sequences with more than 20 representatives in at least one time point are presented. The clones selected for in vivo biodistribution, following expression and stability assays, are depicted.

Following rabbit immunization and validation of the immune response towards brain endothelial cells, our major goal was to select the most proficient antibodies for BBB translocation. Aimed at that objective, a sdAb library was constructed by amplification of the antibody light chain variable regions (V_L_) recovered from the bone marrow and spleen cDNA of the two immunized rabbits. The V_L_ sdAbs regions were then cloned in the pComb3X phagemid vector originating a phage displayed library with a diversity of 1.2 × 10^8^. A major advantage of using sdAb is the possibility of generating large libraries that can be used to screen a diverse set of receptors, enabling the discovery of new targets. A powerful technique for selection of antibodies is phage display. It has been argued that in vivo phage display selection procedures offer an advantage over in vitro screening processes since the antibodies that are displayed at phage surface can be selected in the intricated milieu of the animal based on desired pharmacokinetic and targeting specificity properties [51]. Moreover, antibodies are identified and tested functionally, and must overcome natural barriers and mechanisms of degradation. It is known that in vivo BBB endothelial cells get stimulated by their surrounding cells and intraluminal blood flow. This contributes to the complexity of the BBB and regulates the expression of specific receptors at the cell surface in a polarized fashion. Brought together, these reasons justify that screening for highly specific BBB transmigrating antibodies should preferably be performed in vivo. Thus, to select a panel of rabbit derived brain targeting sdAbs in a natural context, preserving the in vivo BBB characteristics and its innate intracellular interaction with the surrounding cells and intraluminal blood flow [36,37], we performed an in vivo phage display selection as described in the material and methods section and detailed in Figure 4A. Briefly, the phage displayed library (input) was injected in the tail vein of CD1 mice. At 2 or 60 min p.i. the mice were perfused, euthanized, the brains were removed, and phages were recovered to retrieve brain specific sdAbs. The time points were selected based on preliminary assays with a naïve library (data not shown) in order to foster the selection of sdAbs that can swiftly target the BBB with limited brain accumulation features. Two subsequent rounds of selective screenings were performed to enrich the BBB targeting sdAbs population (Figure 4A). At the end of the third round, the phages recovered presented a titer of 10^5^ (phages/mL), reflecting a high enrichment of the phages with increased ability to reach the BBB. In contrast, no enrichment was observed when M13 helper phage was used as a control (Figure 4A). Thus, the implemented in vivo phage display approach enabled the selection of sdAbs in a high stringency and restrictive environment that allowed a simultaneously subtractive selection of non-specific sdAbs. A similar approach was recently published by Stutz and collaborators, where the authors performed an in vivo phage display using a naïve human derived library to identify brain targeting single-chain fragment variable antibodies (scFvs). In this study, the authors recovered two scFvs, namely scFV40 and scFV4, that exhibited binding ability to rat BBB [52]. Yet, in this study a non-immunized derived library was used and the small number of recovered scFvs clearly demonstrates the need of using antibody repertoires from immunized donors and in vivo targeted approaches for discovery of unique and BBB specific targets.

### 3.3. Screening for Antibodies towards the BBB

**Figure 5:**
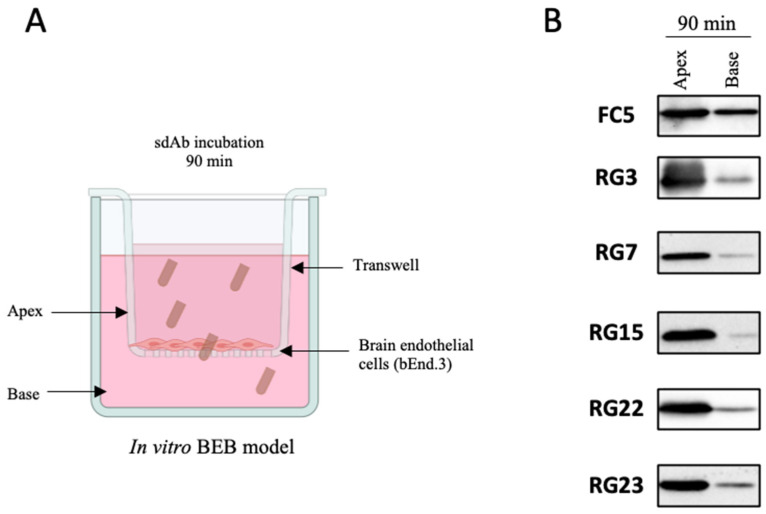
In vitro selection of the dominant sdAbs clones. With the aim of selecting the most competent clones in transposing the BBB, the clones with higher frequency in NGS analysis and proficient in terms of expression and solubility properties (data not shown) were selected for further characterization in terms of in vitro BEB model translocation efficacy. (**A**) 15 μg of each purified sdAb was added to the apex of the transwell and incubated for 90 min. (**B**) Following incubation period, all volumes were collected from the apex and the base and analysed by WB with anti-HA antibody.

To characterize the diversity of the enriched sdAb population and identify the dominant clones, individual colonies recovered from the second and third in vivo selections were randomly picked and analysed by Sanger sequencing. Bioinformatic analysis of 56 sequenced clones revealed the presence of 27 distinct groups, six of which included four or more representatives (data not shown). It was expected that the most prevalent sequences would be repeatedly picked, yet the limitation in terms of simultaneous sequencing process hampers sanger sequencing from giving a wider overview of a 10^5^ phage display library. To get further insights of the enriched sequences we performed next generation sequencing (NGS) of the third biopanning repertoire (Figure 4B). NGS allows massive sequence analysis of the panning population, enabling a genomic assessment of the library diversity and frequency of each clone. Following NGS analysis and subsequent sequencing filtering, we obtained 65,701 sequences from the 2 min and 35,472 sequences from the 60 min selection panning. This decrease of the V_L_ fragment diversity was expected at the 60 min timepoint due to the increased stringency of the selection process. The percentage of singletons (sequences represented by only one count) was similar in both libraries, namely, 42.2% for the 2 min and 46.3% for the 60 min library of the total sequences. Sequence comparison of the main clones recovered from each biopanning demonstrated that the lead sequences were identical in terms of prevalence in both time points, although with a higher occurrence at the 60 min repertoire (N = 5336, representing 15.0% of the total sequences) compared with the 2 min (N = 4548, representing 6.9% of the total sequences) (Figure 4B). Interestingly, among the top sequences from each time point, a higher sequence conservation was observed in the complementary determining regions (CDR), with most differences being allocated mainly at the antibody family level and frameworks (FR) regions (data not shown). Following sequencing analysis, representatives of the most prevalent clones (N = 23) were harvested and characterized in terms of antibody expression and solubility (data not shown). The most stable and predominant clones, highlighted in Figure 4B (N = 5), were chosen for further expression, purification, and characterization in terms of BEB translocation. The ability of individual clones to transpose the BBB was first assessed in the in vitro BEB model. This model allowed us to understand the behavior, stability, and penetrability of each selected sdAbs in a cellular environment (Figure 5A). Briefly, each clone was added to the apical chamber and incubated for 90 min. At the end of each assay, the apical and base volumes were collected and analysed by WB. In the present study a direct comparison of the selected BBB targeting sdAbs was performed with the VHH FC5, a promising camelid antibody selected from a nonimmunized phage library, that targets TMEM30A and has been shown to function as a drug delivery platform [24,32,33]. All selected clones were able to translocate the bEnd.3 cell monolayer as demonstrated in Figure 5B, with clones RG3, RG22 and RG23 showing an increased in vitro BEB-crossing efficiency. This assay allowed us to confirm the selection of highly competent brain targeting and BBB transposing sdAbs.

### 3.4. In Vivo Biodistribution of Selected Clones

A key feature of a potential antibody-based therapy is clearly its targeting ability. This is even more important for CNS approaches due to the intrinsic selectivity of the BBB. With the aim of demonstrating the BBB targeting and translocation ability of the selected antibodies and to determine the brain accumulation of the best clones, ^99m^Tc(CO)_3_–labeled individual sdAbs were intravenously injected in the tail vein of CD1 mice and the ex-vivo radioactivity of individual organs was measured. The biodistribution results of the tested clones at 2 and 60 min p.i. are presented in Table 1 and expressed as percentage of injected activity per gram of organ (% I.A./g ± SD). The total radioactivity excretion is also presented as percentage of the total injected activity (% I.A.). For the sake of comparison, Table 1 includes also the biodistribution profile for the ^99m^Tc(CO)_3_–labeled FC5, a control antibody. Statistical analysis of the results indicated that at 2 min p.i., there was no significant differences between the clones under evaluation and the FC5 in most organs and tissues (blood, intestine, spleen, heart, muscle, bone and stomach) which reflects the short time point after administration. However, significant differences were found in organs related to the excretory paths (liver and kidneys) as well as in the lung and brain uptake. In fact, all tested clones presented a brain accumulation > 0.4% I.A./g at 2 min p.i., with clones RG3 and RG15 displaying the highest brain accumulation and reaching values 0.82 and 0.61% I.A./g, respectively. Both radiolabeled sdAbs predominantly accumulated in the kidneys, which may be related to their preferential renal excretion path and tubular reabsorption. At 60 min p.i., significant differences between the clones and FC5 were found in the radioactivity uptake in all evaluated organs and tissues except in the brain. Significant differences were also found in the rate of total excretion.

As regards to brain uptake, the biodistribution data confirmed a lower accumulation at 60 min p.i., suggesting that sdAbs translocated the BBB and were rapidly recirculated back into the blood and excreted from the animal body and possibly indicating the presence of a receptor on the luminal and basal side of the endothelial cells. This non-retention effect strengthens the potential of the selected sdAbs as drug delivery vectors that may circumvent the brain accumulation issue and its derived toxic effects. Among the selected clones for in vivo testing, four out of the five ^99m^Tc(CO)_3_–labeled sdAbs presented similar or superior brain accumulation compared to FC5. Within the panel of selected sdAbs, a superior brain accumulation was observed for the RG3 clone, (0.82 ± 0.05% I.A./g) and, as far as we are aware, positioning as one of the most competent sdAb in BBB translocation described so far.

### 3.5. Validation of the RG3 as a BBB Drug Delivery Vector

A major goal of our study was also to explore the potential of our selection platform to distinguish nano-antibodies for targeted drug delivery approaches. To achieve this, the most competent sdAb, RG3, along with the control FC5, were engineered at liposome surface encapsulating the HDACi PAN, and its BBB-transmigrating properties and antitumoral activity were validated in a dual functional in vitro BEB-glioblastoma model. Glioblastoma is an aggressive brain tumor highly resistant to chemotherapy, with very limited therapeutic strategies due to the poor drug penetration through the BBB. Among the promising strategies for cancer treatment, PAN has emerged as a highly efficient new class of anticancer drug. Indeed, its promising anticancer properties have been demonstrated for different types of cell lines including lymphoma [53], multiple myeloma [54], and diffuse intrinsic pontine glioma [55], being in clinical trials for recurrent glioblastoma [56,57]. Nevertheless, PAN, as many other chemotherapeutics, does not cross the BBB [58]. For this reason, targeted nanoparticles are an appealing tool to improve its delivery into the CNS. Indeed, as reviewed by Hersh and collaborators [59], a variety promising approaches based on different nanoparticle structures functionalized with different targeting ligands have been developed to function as brain shuttles.

Among the wide panel of nanoparticles, liposomes are on the front line of nanocarrier based strategies for glioma therapy [60]. Liposomes are lipid vesicles constituted by one or more concentric lipid bilayers separated by aqueous compartments. Due to their unique characteristics, incorporation of both hydrophobic and hydrophilic compounds, along with its biocompatibility, prolonged circulation time, sustained drug delivery and ability to be conjugated with a targeting moiety, liposomes have a remarkable potential as brain-targeted carrier systems [60,61]. In addition, the nine FDA approved liposomal based formulations for cancer therapy further supports the therapeutic potential of this lipid base carriers [60]. Therefore, liposomes encapsulated with PAN and conjugated with our BBB-sdAbs are a promising approach to validate the proof-of-concept of our novel targeted drug delivery system.

**Figure 6:**
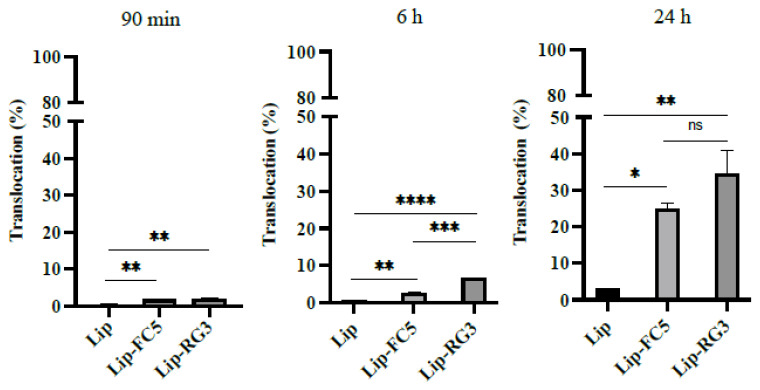
Translocation of RG3 and FC5 functionalized liposomes in the in vitro BEB model (Lip-RG3 and Lip-FC5). To determine the effect of RG3 functionalization in liposome in vitro BEB translocation, rhodamine loaded, biotinylated liposomes were surface modified with RG3 and added to the apex of the transwell, as described previously, and incubated for 90 min, 6 h and 24 h. The translocation percentage was determined by measuring the rhodamine fluorescence intensity at the base. Non functionalized liposomes (Lip) and FC5 functionalized liposomes (Lip-FC5) were used as controls. A one-way ANOVA statistical test followed by a Turkey’s test was used to compare each liposomal formulations (**** *p* < 0.0001; *** *p* < 0.001; ** *p* < 0.01; * *p* < 0.05; ns, not statistically significant).

In our study, liposomes loaded with PAN were successfully developed, with a mean size of 110 nm and an encapsulation efficiency of 65 ± 2% using the lipid composition DPPC:Chol:DSPE-PEG:DSPE-PEG-Biotin at a molar ratio of 1.85:1:0.14:0.01. No significative differences in terms of encapsulation efficiencies and vesicle sizes were observed among biotinylated and non-biotinylated liposomes. To assess the cellular cytotoxicity of the unconjugated and BBB-sdAbs conjugated PAN-loaded liposomes against glioblastoma, a cell viability assay was carried out with the glioblastoma cell line LN229 (Figure S1). Similar to the free PAN formulation, PAN loaded liposomes exhibited a potent activity and dose-dependent inhibitory effect on the proliferation of LN229 cells (Figure S1A). Moreover, the antibody conjugation at liposomes surface did not affect the inhibitory effects of PAN loaded liposomes on the proliferation of LN229 cells and no significant differences between liposomes conjugated with FC5 or RG3 were observed (Figure S1B). In contrast, no cytotoxicity activity was observed for PAN free RG3 and FC5 liposome formulations (Lip-RG3 and Lip-FC5, Figure S1C). In parallel, we evaluated the translocation efficiency of the Lip-RG3 and Lip-FC5 in the in vitro monocellular model. Here, RG3 and FC5 functionalized rhodamine loaded liposomes were added to the apical side of the model and collected following a 90 min, 6 and 24 h incubation, reaching a translocation maximum at the model base of 29.3 ± 6.5% at 24 h in the case of RG3-liposome, 9.4 times higher than the non-conjugated liposomal formulation. In turn, the percentage of translocation for the FC5 conjugated liposome was 12.9 ± 1.6%, only 4.2 times higher than the non-conjugated liposomal formulation (Figure 6). In addition, monitorization of BEB integrity demonstrated that both liposomal formulations had no significant impact at the barrier structure (Table S1).

A similar study was performed by Lakkadwala and collaborators, which developed liposomes encapsulated with doxorubicin and erlotinib, functionalized with transferrin (Tf) and a cell penetrating peptide (TAT or QLPVM). The highest in vitro translocation was observed for liposomes with Tf and TAT dual functionalization, reaching 12.72% at the base of the co-culture system [62]. Despite being a dual system with Tf and TAT, our Lip-RG3 demonstrated a higher translocation efficiency at 24 h incubation (29.3%).

**Figure 7:**
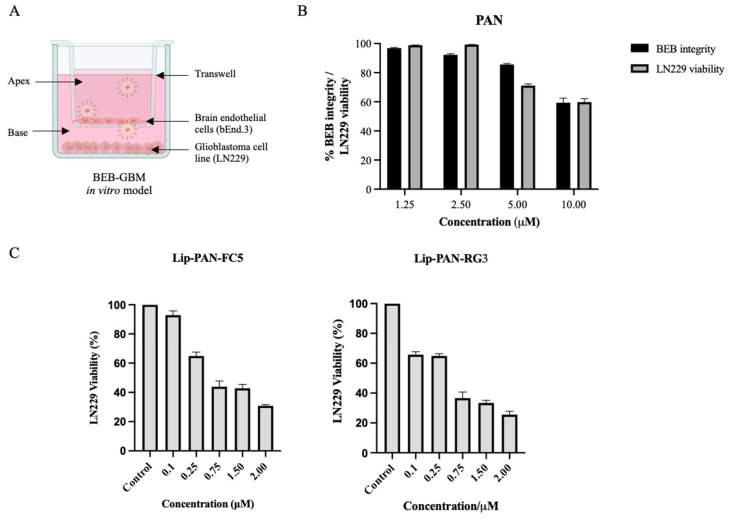
Activity of liposomal drug delivery in the BEB-Glioblastoma model. (**A**) An in vitro BEB-GBM model was developed to mimic the complexity of drug delivery to a brain tumor. This model combined a monolayer of bEnd.3, as the BBB barrier, and the LN229 cell line at the base as the glioblastoma tumour. (**B**) To evaluate the ability of free PAN to translocate the BEB and its effect on the integrity of the model barrier, PAN was added at increasing concentrations to the apex of the BEB-GBM model and incubated for 24 h. The integrity of the bEnd.3 was measured by determining the translocation of the FD40 fluorescent probe while LN229 viability was determined using WST-1 reagent and OD_450nm_ measurement following 24 h incubation. (**C**) PAN encapsulated RG3 and FC5 functionalized liposomes were analyzed in terms of BEB translocation and delivery of PAN to a glioblastoma cell line and compared with non-functionalized unloaded liposomes (Control). Briefly, PAN loaded liposomes were added to the apex of the BEB-GBM model and incubated for 24 h. The cytotoxic effect of PAN in LN229 was determined by adding WST-1 reagent. The values were obtained from duplicates of two independent experiments.

Finally, the antiproliferative activity of the PAN loaded in RG3 and FC5 conjugated liposomes following BBB translocation was determined in a dual functional in vitro BEB-glioblastoma model (BEB-GBM) (Figure 7). To develop the BEB-GBM model, a non-contact co-culture system composed of bEnd.3 as the BBB barrier and the LN229 cell line at the base as the GBM cell line was implemented (Figure 7A). To evaluate the cytotoxicity on bEnd.3 cells and determine the most appropriate PAN concentration to be tested in the BEB-GBM model, increasing concentrations of free PAN were added to the apex of the BEB-GBM model and its effect in the BEB integrity and LN229 cytotoxicity was determined. As shown in Figure 7B, no significant effect in the BEB integrity and LN229 viability was observed when PAN free was added below 2.5 µM. Thus, the BEB translocation efficiency and drug delivery of the PAN encapsulated in RG3 and FC5 functionalized liposomes was assessed by adding liposomes to the BEB-GBM model apex following 24 h incubation at different concentrations (0.1–2.0 µM). As shown in Figure 7C, both RG3 and FC5 functionalized PAN liposomes were able to translocate the in vitro BEB-GBM model and exhibited a dose-dependent inhibitory effect in the LN229 cell line without influencing the overall barrier integrity of the monocellular model (Supplementary Material Table S1). A similar study was performed by Mu and collaborators. The authors developed vinblastine liposomes functionalized with a transferrin targeting molecule, the TfR-T12, to potentiate the translocation of vinblastine across the BBB and eliminate brain glioma [63]. As in the present study, the authors validated the BBB targeted lipid base nanoparticle in an in vitro model and observed a significant killing effect against the glioma cell line, further supporting the drug delivery strategy.

To corroborate that the cytotoxic effects of the RG3 and FC5 conjugated liposomes on GBM cell line were related to histone acetylation induction, the key molecular mechanism of HDACis, the H3 acetylation status of cells treated with PAN-loaded in RG3 and FC5 conjugated liposomes was compared with the H3 acetylation status of unloaded liposome formulations and vehicle/control treated cells (Figure S2). Immunoblotting analysis demonstrated that LN229 GBM cell line presented an hyperacetylation status following 24 h treatment with both RG3 and FC5 PAN-loaded liposomes, when compared with unloaded liposomes and vehicle/control treated cells. A final in vivo proof-of-concept biodistribution study was performed to demonstrate the BBB translocation of our RG3 functionalized liposome system. For that, ^111^indium radiolabeled RG3 functionalized liposomes were prepared and administered to CD1 mice. As shown in Table 2, 2 min after intravenous injection (i.v.), 1% of the injected dose per gram of tissue (%ID/g), of our developed radiolabeled system, has reached the brain and this amount was constant 60 min after administration. In contrast, a significantly lower brain uptake was observed for the unconjugated liposome. Statistical analysis of the biodistribution and excretion data at 1 and 24 h p.i., between the RG3-conjugated liposomes and the control liposomes indicate significant differences in almost all studied organs and tissues at 1 h p.i., except the intestines and stomach. Indeed, a significantly higher activity was found in the blood stream and a higher uptake in most organs. A significantly lower rate of total radioactivity excretion of the RG3 conjugated liposomes (15.4 ± 2.5% and 28.2 ± 2.7% I.A. at 1 h and 24 h, respectively versus 59.7 ± 6.6% and 68.3 ± 0.4% for the control liposomes). At 24 h p.i. the significant differences were maintained for the main organs namely blood, brain, excretory organs (kidneys, liver, intestines) and total radioactivity excretion. Altogether, the in vivo and in vitro data have shown that our selected BBB-sdAbs and the developed sdAb-liposome system have a high ability to cross the BBB and are a promising strategy for CNS-targeted therapies.

## 4. Conclusions

To sum up, in the present study we demonstrate that with an immunized rabbit derived sdAb library developed towards BBB epitopes and an in vivo phage display selection method, a panel of nano-antibodies with one of the highest levels of BBB translocation described so far was identified. Moreover, the developed RG3-liposome specifically targets and translocates the BBB, delivering a payload, in in vitro settings, at effective concentrations and constituting a strong candidate for drug delivery to the CNS. Further studies regarding the identification of the receptors targeted by the selected sdAbs are ongoing which will allow a better elucidation of the transport mechanism. Hence, our proposed in vivo sdAb development platform is a pioneering selection process of highly specific nano-antibodies with promising properties for brain targeting and drug delivery to different CNS diseases, such as brain tumors, Alzheimer’s, or Parkinson diseases.

## 5. Patents

The work reported in this article resulted in the submission of a provisional national patent with number 117330. FAS, SIA, JNRD and SO are inventors of the patent. Patent rights granted to Technophage SA and Faculdade de Medicina Veterinária Universidade de Lisboa.

## Figures and Tables

**Table 1:** Biodistribution profiles of ^99m^Tc(CO)_3_-labeled sdAbs. To validate that the selected clones from phage display selection were the most competent sdAbs in terms of BBB translocation, an in vivo biodistribution assay was performed. SdAbs were radiolabeled with ^99m^Tc(CO)_3_(H_2_O)_3_ and intravenously injected in the tail vein of CD1 mice. Mice were sacrificed by cervical dislocation at 2 and 60 min p.i. and the radioactivity of each organ measured in a dose calibrator. The uptake in the brain and tissues of interest was calculated and expressed as a percentage of injected activity per gram of tissue (%I.A./g). Total radioactivity excretion was expressed as percentage of injected activity (%I.A.). Statistical analysis was performed with one-way ANOVA, with *p*-value < 0.05 considered as the level of statistical significance.

Organ	RG3-^99m^Tc		RG7-^99m^Tc		RG15-^99m^Tc		RG22-^99m^Tc		RG23-^99m^Tc		FC5-^99m^Tc	
	2 min	60 min	2 min	60 min	2 min	60 min	2 min	60 min	2 min	60 min	2 min	60 min
Blood	10.1 ± 2.6	1.4 ± 0.1	11.2 ± 2.4	1.5 ± 0.6	12.5 ± 2.4	3.0 ± 0.1	13.2 ± 2.5	0.8 ± 0.1	7.8 ± 0.2	1.1 ± 0.8	11.0 ± 2.4	1.0 ± 0.20
Liver	5.0 ± 0.9	1.9 ± 0.3	3.8 ± 0.8	1.0 ± 0.4	6.0 ± 1.0	3.6 ± 0.7	4.6 ± 0.7	1.7 ± 0.2	5.3 ± 0.2	1.9 ± 0.4	10.0 ± 1.5	12.40 ± 0.4
Intestine	1.8 ± 0.6	0.7 ± 0.1	1.8 ± 0.2	0.21 ± 0.08	1.8 ± 0.4	0.4 ± 0.1	2.0 ± 0.2	0.17 ± 0.03	1.6 ± 0.7	0.4 ± 0.1	0.9 ± 0.4	0.79 ± 0.04
Spleen	2.2 ± 0.6	0.6 ± 0.1	3.6 ± 0.6	0.5 ± 0.3	3.5 ± 1.2	4.8 ± 1.7	2.6 ± 0.6	0.54 ± 0.03	2.45 ± 0.04	0.8 ± 0.1	4.2 ± 0.8	4.2 ± 0.80
Heart	3.2 ± 1.2	0.4 ± 0.1	4.7 ± 1.2	0.45 ± 0.08	4.3 ± 0.8	1.3 ± 0.2	3.2 ± 0.5	0.29 ± 0.03	4.5 ± 0.4	0.5 ± 0.1	3.9 ± 0.5	0.76 ± 0.03
Lung	7.5 ± 2.7	1.1 ± 0.2	9.8 ± 2.4	1.5 ± 0.7	9.9 ± 1.2	2.4 ± 0.4	6.7 ± 0.6	2.1 ± 0.4	6.0 ± 0.9	1.7 ± 0.3	12.6 ± 0.6	38.20 ± 6.20
Kidney	74.6 ± 9.9	170.6 ± 19.1	73.6 ± 7.5	93.1 ± 6.9	54.6 ± 7.5	52.1 ± 9.0	55.5 ± 0.9	49.3 ± 8.3	27.2 ± 4.1	74.3 ± 10.7	59.8 ± 2.4	96.40 ± 6.80
Muscle	2.0 ± 1.0	0.4 ± 0.2	1.9 ± 0.2	0.29 ± 0.07	1.5 ± 0.4	0.6 ± 0.1	1.4 ± 0.1	0.3 ± 0.1	1.5 ± 0.2	0.34 ± 0.05	1.1 ± 0.1	0.39 ± 0.04
Bone	2.7 ± 0.2	0.81 ± 0.05	2.3 ± 0.4	0.33 ± 0.05	2.3 ± 0.8	1.0 ± 0.1	2.4 ± 0.4	0.5 ± 0.1	1.5 ± 0.4	0.4 ± 0.1	1.7 ± 0.2	0.80 ± 0.3
Stomach	1.0 ± 0.4	0.97 ± 0.04	1.7 ± 0.6	0.9 ± 0.4	0.7 ± 0.3	1.2 ± 0.1	0.7 ± 0.3	0.26 ± 0.07	1.1 ± 0.5	0.9 ± 0.4	1.2 ± 0.2	0.80 ± 0.3
Brain	0.82 ± 0.05	0.06 ± 0.02	0.46 ± 0.1	0.4 ± 0.1	0.61 ± 0.1	0.08 ± 0.06	0.60 ± 0.15	0.03 ± 0.01	0.49 ± 0.02	0.04 ± 0.02	0.52 ± 0.18	0.07 ± 0.02
Excretion (%I.A.)	-	19.6 ± 5.4	-	21.1 ± 9.0	-	27.5 ± 5.1	-	70.3 ± 3.5	-	27.5 ± 5.1	-	9.60 ± 1.40

**Table 2:** Biodistribution of selected RG3 conjugated liposome: to validate the BBB translocation of the RG3-conjugate liposome an in vivo biodistribution assay was performed. RG3-conjugated liposomes were radiolabeled with ^111^In and intravenously injected of the tail vein of CD1 mice. Mice were sacrificed by cervical dislocation at 2 min, 60 min and 24 h following injection and the radioactivity of each organ measured using a dose calibrator. The uptake in the brain and tissues of interest was calculated and expressed as a percentage of injected radioactivity dose per gram of tissue (%ID/g). Total radioactivity excretion was expressed as percentage of injected activity (%I.A.). Statistical analysis of the data was performed (*t*-test), with *p*-value <0.05 considered as the level of statistical significance.

Organ	^111^In-Lip-RG3			^111^In-Lip-(Control)	
	2 min	60 min	24 h	60 min	24 h
Blood	33.0 ± 7.4	26.2 ± 8.1	8.9 ± 1.7	12.7 ± 3.8	3.5 ± 0.6
Liver	8.1 ± 2.8	10.7 ± 2.1	11.7 ± 1.6	1.1 ± 0.6	5.9 ± 0.5
Intestine	0.4 ± 0.1	0.9 ± 0.4	2.3 ± 0.1	0.55 ± 0.06	0.98 ± 0.07
Spleen	4.3 ± 0.8	19.4 ± 3.2	18.2 ± 0.1	1.7 ± 0.4	12.5 ± 4.2
Heart	4.2 ± 0.8	4.4 ± 1.2	3.0 ± 1.3	0.7 ± 0.2	1.3 ± 0.3
Lung	13.3 ± 3.9	10.2 ± 1.0	3.9 ± 1.6	1.0 ± 0.5	1.6 ± 0.6
Kidney	7.0 ± 1.7	6.6 ± 1.0	6.4 ± 0.7	1.3 ± 0.6	2.7 ± 0.3
Muscle	0.8 ± 0.2	0.62 ± 0.07	0.9 ± 0.2	0.4 ± 0.1	1.3 ± 0.9
Bone	1.8 ± 0.1	1.6 ± 0.2	1.1 ± 0.9	0.6 ± 0.3	0.7 ± 0.3
Stomach	0.8 ± 0.2	0.8 ± 0.6	0.8 ± 0.2	1.7 ± 0.9	1.1 ± 0.2
Brain	1.0 ± 0.3	1.0 ± 0.4	0.4 ± 0.2	0.13 ± 0.03	0.16 ± 0.07
Carcass (%I.A.)	45.2 ± 1.8	43.6 ± 4.8	38.3 ± 3.6	18.0 ± 0.9	26.0 ± 2.3

## Data Availability

The data that supports this study finding are available from the corresponding author, upon reasonable request.

## Supplementary Materials

The following are available online at https://www.mdpi.com/article/10.3390/pharmaceutics13101598/s1, Figure S1: Cytotoxicity assay of study formulations in LN229 cells. LN229 cells (20 × 10^4^) were subjected to increasing concentrations of PAN and Lip-PAN (A) and Lip-PAN-RG3 and Lip-PAN-FC5 (B) to determine the cytotoxicity of PAN in the different formulations. As a control, the effect of empty liposomes was also determined (C). After 24 h treatment, WST-1 reagent was used to determinate cell viability and proliferation. Three replicates were used to determinate each data point and two independent experiments were performed in different days, Figure S2: Cytotoxicity effects of immunoliposomes encapsulated with panobinostat in histone acetylation. LN229 cells were exposed to 2.5 μM of encapsulated panobinostat. After 24 h treatment in the BEB glioblastoma model cells were harvest with RIPA buffer for total protein extraction. Acetylation of H3 histones were evaluated by WB using anti-acetyl-histone H3 polyclonal antibody. DMEM medium was used as vehicle control. Anti-histone H3 antibody was performed to control loading, Table S1: Integrity of BEB in vitro model, after translocation with non-functionalized and FC5 and RG3 functionalized liposomes at 90 min, 6 h and 24 h incubation.
