# Supplementary material for: Highly Specific Blood-Brain Barrier Transmigrating Single-Domain Antibodies Selected by an In Vivo Phage Display Screening
Source: Pharmaceutics. 2021 Oct 2;13(10):1598. doi: 10.3390/pharmaceutics13101598 (PMC8540410; doi:10.3390/pharmaceutics13101598)
Supplement: Supplementary file 1 [file pharmaceutics-13-01598-s001.zip › pharmaceutics-1351891-supplementary.pdf]

pharmaceutics

Supplementary Material: Highly Specific Blood-Brain Barrier Transmigrating Single-Domain Antibodies Selected by an In Vivo Phage Display Screening

Sandra Isabel Aguiar, Joana N. R. Dias, Ana Santos André, Marta Lisete Silva, Diana Martins, Belmira Carrapiço, Miguel Castanho, João Carriço, Marco Cavaco, Maria Manuela Gaspar, Rui Jorge Nobre, Luís Pereira de Almeida, Soraia Oliveira, Lurdes Gano, João D. G. Correia, Carlos Barbas 3rd, João Gonçalves, Vera Neves and Frederico Aires-da-Silva

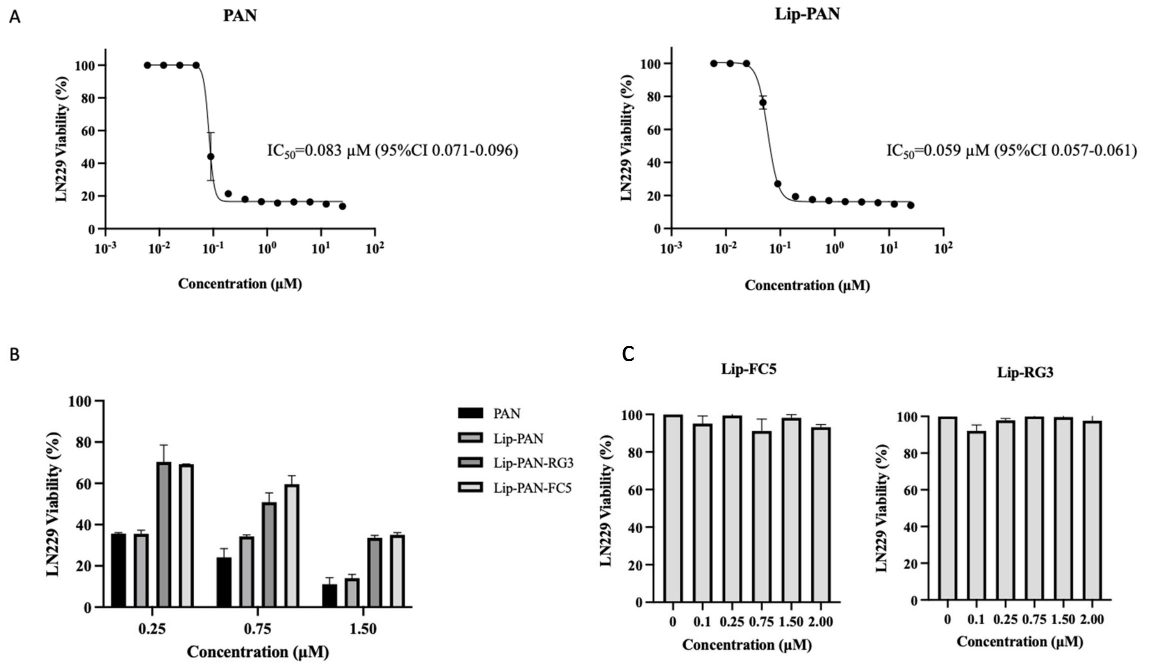

Figure S1. Cytotoxicity assay of study formulations in LN229 cells. LN229 cells (20x10[4]) were subjected to increasing concentrations of PAN and Lip-PAN (A) and Lip-PAN-RG3 and Lip-PAN-FC5 (B) to determine the cytotoxicity of PAN in the different formulations. As a control, the effect of empty liposomes was also determined (C). After 24h treatment, WST-1 reagent was used to determinate cell viability and proliferation. Three replicates were used to determinate each data point and two independent experiments were performed in different days.

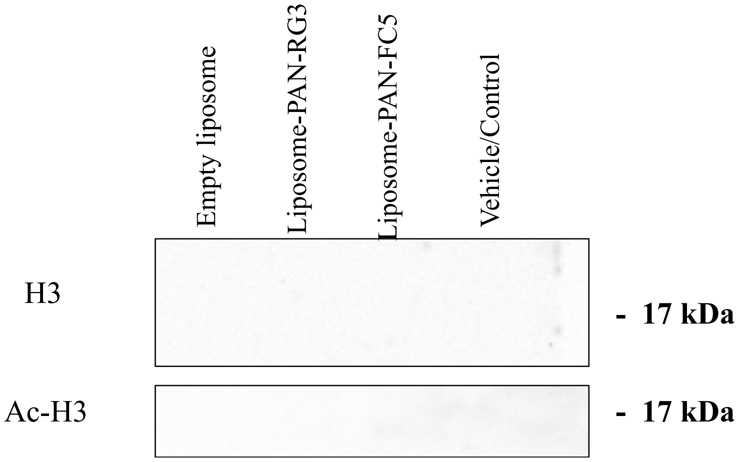

Figure S2. Cytotoxicity effects of immunoliposomes encapsulated with panobinostat in histone acetylation. LN229 cells were exposed to 2.5 µM of encapsulated panobinostat. After 24 h treatment in the BEB glioblastoma model cells were harvest with RIPA buffer for total protein extraction. Acetylation of H3 histones were evaluated by WB using anti-acetyl-histone H3 polyclonal antibody. DMEM medium was used as vehicle control. Anti-histone H3 antibody was performed to control loading.

Table S1. Integrity of BEB *in vitro* model, after translocation with non-functionalized and FC5 and RG3 functionalized liposomes at 90 min, 6 h and 24 h incubation.

Sample	BBB Integrity (%)
Positive Control	90.4 ± 1.0
Lip (90 min)	94.7 ± 1.0
Lip-FC5 (90 min)	90.6 ± 0.9
Lip-RG3 (90 min)	87.9 ± 0.5
Lip (6h)	87.4 ± 0.3
Lip-FC5 (6h)	95.8 ± 2.9
Lip-RG3 (6h)	90.7 ± 0.6
Lip (24h)	95.2 ± 1.0
Lip-FC5 (24h)	92.2 ± 3.0
Lip-RG3 (24h)	95.2 ± 1.5
